# NECT Is Next: Implementing the New Drug Combination Therapy for *Trypanosoma brucei gambiense* Sleeping Sickness

**DOI:** 10.1371/journal.pntd.0000720

**Published:** 2010-05-25

**Authors:** Oliver Yun, Gerardo Priotto, Jacqueline Tong, Laurence Flevaud, François Chappuis

**Affiliations:** 1 Médecins Sans Frontières/Doctors Without Borders, New York, New York, United States of America; 2 Epicentre, Paris, France; 3 Médecins Sans Frontières, Geneva, Switzerland; 4 Médecins Sans Frontières, Barcelona, Spain; 5 Geneva University Hospitals, Geneva, Switzerland; Institute of Tropical Medicine, Belgium

## Introduction

Fatal if untreated, human African trypanosomiasis (HAT; sleeping sickness) afflicts an estimated 50,000–70,000 people each year [1], all in sub-Saharan Africa, with only a minority of cases (nearly 12,000 in 2008) being reported [2]. HAT is one of four neglected tropical diseases (NTDs) identified by the World Health Organization (WHO) as requiring Innovative and Intensified Disease Management (IDM), along with Chagas disease, leishmaniasis, and Buruli ulcer [3]. These particular NTDs have poorly understood burdens, lack optimal control tools, receive insufficient research and development (R&D) investment, and affect people who often live in remote or insecure areas with limited access to health care. Excluding Buruli ulcer, these IDM diseases have the highest death rates of all NTDs [4].

HAT in west and central Africa is caused by the protozoan parasite *Trypanosoma brucei gambiense*, transmitted through tsetse flies. The disease progresses from first stage (infecting blood and lymph) to second stage (infecting the central nervous system), which can lead to severe sleep disturbances, neurological and psychiatric disorders, coma, and death. Primary elements of HAT management are surveillance, diagnosis, treatment, and vector control.

Drug treatments for *T. b. gambiense* HAT have been limited: pentamidine for first-stage disease, and melarsoprol or eflornithine for second-stage disease. Eflornithine is safer and often more effective than melarsoprol, which is associated with high toxicity, even fatal at times, and exhibits high rates of treatment failure in numerous HAT-endemic foci. However, despite an increasing proportion of second-stage HAT treated with eflornithine during recent years [5], melarsoprol remains in use in many treatment centers due to eflornithine's long, burdensome treatment administration requirements, which are difficult to implement in resource-constrained settings.

In April 2009, a new treatment option, nifurtimox-eflornithine combination therapy (NECT), was added to the WHO Essential Medicines List (EML) for the treatment of second-stage *T. b. gambiense* HAT [6]. NECT was added to the EML based on the high efficacy and good safety profile observed in all studies done to date, against a background of recognized severity of stage 2 disease and toxicity of existing treatments. Surveillance of adverse events was strongly recommended [7]. Compared with eflornithine monotherapy, NECT is easier to administer and requires fewer human and material resources. In the current context, NECT stands as the most promising first-line treatment for second-stage *T. b. gambiense* HAT. Here we describe the developments and challenges in rolling out and implementing NECT in HAT-endemic areas.

## NECT Development

### History

In response to the lack of new drug entities for HAT treatment, and the inadequate and undesirable features of existing drugs, new alternative therapies needed to be assessed. Based on the known utility of combination therapies in attenuating toxicity, maintaining or increasing efficacy, and preventing resistance, this avenue was explored by evaluating drug combinations including eflornithine and melarsoprol, along with nifurtimox, a drug used to treat another trypanosomal illness, Chagas disease (American trypanosomiasis), and shown to have varying efficacy against HAT [8]–[10].

From 2001 through 2004, Epicentre, the research and epidemiology arm of Médecins Sans Frontières/Doctors Without Borders (MSF), conducted two sequential clinical drug-combination studies at HAT treatment sites in northern Uganda, which revealed the potential of the nifurtimox-eflornithine combination as a highly effective and well-tolerated therapy [11], [12]. Based on these initial studies, Epicentre and MSF launched in the Republic of Congo (RoC) a demonstration trial comparing this therapy to the best available therapy at the time, intravenous eflornithine for 14 days [13].

This study was completed through a multicentric extension in the Democratic Republic of Congo (DRC), in collaboration with the countries' ministries of health (MOHs), the Drugs for Neglected Diseases initiative (DNDi), and the Swiss Tropical Institute (STI; now known as Swiss Tropical and Public Health Institute [Swiss TPH]). The whole multicentric study extended from 2003 through 2008. The combination of nifurtimox and eflornithine was found to be a marked improvement for second-stage HAT therapy, with key advantages over the previous therapeutic options [14], [15].

### Advantages

In the randomized, open-label, phase III trial at four centers in DRC and RoC, NECT was shown to be easier to administer than, and noninferior in efficacy to, eflornithine monotherapy for the treatment of second-stage *T. b. gambiense* HAT [14]. The drug combination was fairly well tolerated: patients treated with NECT had half as many major drug-related adverse events as those treated with eflornithine alone (14% versus 29%; *P* = 0.002). The noninferiority in efficacy of NECT versus eflornithine monotherapy (as measured by 10% difference in cure rates) was demonstrated by 96.5% cure rate for NECT group versus 91.6% for eflornithine group in the intention-to-treat patient population, and 97.7% versus 91.7% in the per-protocol population, both at 18 months follow-up.

While eflornithine monotherapy requires 56 intravenous (IV) infusions over 14 days, NECT requires only 14 infusions over 7 days (plus oral nifurtimox 3 times per day for 10 days). NECT's shorter treatment duration and considerably fewer IV infusions make its administration less difficult and cumbersome for both the patients and care providers.

The cost of NECT kits (supplies and preparation time; excluding the cost of the drugs, which are donated) is €39 per patient, compared with €107 per patient for eflornithine monotherapy kits (unpublished data, MSF-Logistique, February 2010). This large cost difference is due to fewer quantities of drugs, injection fluids, and other materials, resulting in less volume and weight to transport (four NECT treatments per kit, compared with two eflornithine monotherapy treatments per kit). Cost differences may be even larger when taking into account indirect expenditures such as shorter lengths of hospital stay, transport of lighter kits to the endemic country's capital and from the capital to the field, and management of fewer adverse events.

When comparing the cost of NECT against melarsoprol, a simple cost comparison would be inappropriate because of melarsoprol's high toxicity and declining effectiveness. A cost-effectiveness study showed that the cost per life saved was similar between melarsoprol and standard eflornithine monotherapy [16]. It is therefore reasonable to assume that NECT's cost per life saved will be lower than that of melarsoprol, though this requires further study.

As a combination of drugs with different modes of action, NECT also has less potential for emergence of parasitic resistance, which is a major drawback of long-term use of monotherapies, as shown with melarsoprol [17].

### New Research

Further data on the safety, effectiveness, and feasibility of NECT are expected from the NECT-FIELD study, which commenced April 2009 and is currently recruiting patients [18]. This multicenter, open-label phase IIIb study is being carried out by DNDi in association with Swiss TPH and the national HAT control program of DRC, Programme National de Lutte contre la Trypanosomiase Humaine Africaine (PNLTHA). An estimated 620 patients will be treated with NECT under field conditions at regular treatment centers in DRC run by PNLTHA and nongovernmental organizations.

## Implementing NECT in the Field

### Recent and Ongoing Developments

The addition of NECT to the WHO EML in April 2009 has paved the way for its rollout and implementation in affected countries. NECT is provided free of charge by WHO through MSF-Logistique, the logistics and supplies division of MSF. Because nifurtimox is not registered for use for HAT, the WHO first requires country MOHs to sign disclaimer letters, in which the MOH takes legal responsibility for the off-label use of the drug.

Despite initial fears that this disclaimer letter prerequisite could present an obstacle to NECT use [19], the MOHs of Central African Republic (CAR), Chad, DRC, Equatorial Guinea, south Sudan, and Uganda have signed the letters at the time of this writing. Other countries have expressed the same intentions and appear to be close to signing soon, including Angola and RoC. These seven countries (excluding Equatorial Guinea) have the highest burdens of HAT, reporting 98.8% of all cases of *T. b. gambiense* HAT in 2006 [2]. Country-level acceptance of NECT has therefore been positive, and acceptance by other countries where HAT is present should translate into concrete, rapid improvement in the field. Physician and patient acceptance of NECT is also important and should be followed.

With disclaimer letters signed, MOH requests to WHO for NECT drugs and supplies have begun. For example, DRC ordered and received 1,000 NECT kits in November 2009, with more orders placed since, and CAR ordered and received 250 kits.

**Figure 1:**
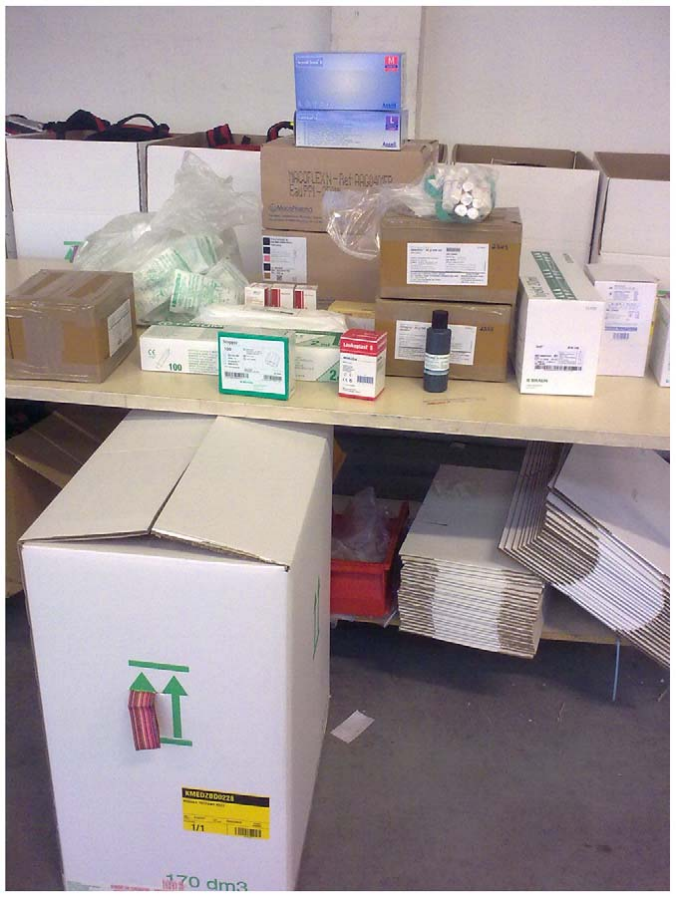
Preparation of a nifurtimox-eflornithine combination therapy (NECT) kit at MSF-Logistique, Mérignac, France. Photo credit: V. Carlier/MSF-Logistique.

MSF-Logistique assembles and ships the NECT kits from its headquarters in Mérignac, France, near Bordeaux (Figure 1). The kits, designed in collaboration between MSF-Logistique and WHO, include all the drugs, fluids, and medical materials for the treatment protocol. The drugs are donated by the manufacturers. In September 2009, Bayer agreed to donate 400,000 tablets of nifurtimox per year to WHO through 2014. Aventis and later sanofi-aventis have donated eflornithine to WHO through two consecutive 5-year agreements since 2001. Kits are being made available free of charge to countries by WHO, with financial support from sanofi-aventis covering the costs of materials and transport to the capital of each country. Each 41-kg kit contains four full treatments of NECT. The volume per NECT treatment is reduced by more than half compared to eflornithine monotherapy.

The first WHO-sponsored medical training session for administering NECT was held in November 2009 in Kinshasa, DRC, for French-speaking countries, with ten representatives from Cameroon, CAR, Chad, DRC, Guinea, and RoC. Another NECT training session took place in February 2010 in Omugo, Uganda, for Uganda and south Sudan. More training modules are planned in 2010 in French, Portuguese, and English.

### Current and Future Challenges

Wide-scale delivery of NECT faces a number of challenges, some specific to NECT, and others related to HAT treatment and control in general.

#### Getting rid of melarsoprol as first-line treatment of stage 2 *T. b. gambiense* HAT

One of the key challenges for NECT implementation is to replace the use of melarsoprol with NECT as first-line treatment for second-stage *T. b. gambiense* HAT. A derivative of arsenic and highly toxic, melarsoprol use is associated with frequent serious adverse events and unacceptably high case-fatality rates. Nevertheless, melarsoprol remains widely used for second-stage *T. b. gambiense* HAT where eflornithine is not available or practical [20]. According to a 2008 assessment of eight provinces in DRC, 50% of patients with second-stage HAT were still being treated with melarsoprol (with the other half treated with eflornithine monotherapy) (unpublished data, PNLTHA). Alarmingly, in one district, Bandundu, which had the heaviest HAT caseload of the provinces surveyed, 96% of second-stage HAT patients were still treated with melarsoprol.

Melarsoprol injections are often painful for patients. Severe adverse events are frequently associated with its use, particularly the development of reactive encephalopathy in 5%–10% of patients, of whom up to 70% die [21]–[24]. Treatment failure with melarsoprol is also a serious concern in various disease foci in several countries, with reports of relapse rates up to 59% [25]–[27]. Treatment failures include relapse (or probable relapse), lack of response to treatment, or death. These failures suggest the emergence of parasitic resistance to melarsoprol [17].

Donors, policymakers, and national programs should now aim for the withdrawal of melarsoprol as first-line treatment for second-stage *T. b. gambiense* HAT with the shortest possible delay. In endemic areas where treatment with melarsoprol is still predominant, NECT protocol change and training should be prioritized. Country-by-country analyses and forecasts will be needed to assess NECT implementation, with comparisons to melarsoprol use. The use of melarsoprol should soon be restricted to treat relapses of *T. b. gambiense* HAT after initial first-line treatment with NECT or eflornithine, and to treat second-stage HAT due to *T. b. rhodesiense*.

#### Transport and supply

Logistical difficulties of getting NECT kits to the field are a concern. The timely transport of treatment kits within endemic countries, from the capital to the hospitals and clinics in the field, remains a common bottleneck.

Drug supply and access are perpetual issues for NTD treatment programs. The donations of nifurtimox and eflornithine from the drug manufacturers are most welcome and must be sustained for NECT to be widely implemented.

#### Training

Although relatively simpler and safer than the older HAT treatment protocols, the training needs for NECT are still considerable in treatment centers that have not yet used eflornithine. Care providers must be trained in the correct nursing care of IV catheters, precise and time-dependent IV administration of eflornithine, daily oral administration of nifurtimox under surveillance (directly observed treatment [DOT]), monitoring of adverse events, and follow-up. DOT is important to ensure treatment adherence in patients who are often mentally disturbed (due to the neurological effects of stage 2 infection), in a low educational level context, and/or at risk of vomiting the tablets. Less-intense training is needed in places where eflornithine monotherapy has already been introduced, since the NECT protocol is similar but simpler.

#### Vertical approaches still needed in some areas

Current NTD donor and policy discussions include a strong focus on program integration into existing primary health care structures [28]. Integration may indeed be ideal for control of NTDs, including for HAT. However, in practice this “one size fits all” strategy may not be feasible for HAT given the complex heterogeneity of its epidemiology and the lack of appropriate diagnostic and treatment tools. Many HAT-endemic areas are in remote, rural areas or in regions of conflict and insecurity, with little or no health infrastructure in which to integrate.

In these contexts, obstacles to HAT diagnosis and treatment, including integration into primary health care systems, are therefore expected. One major hurdle lies in the complexity and sophistication of HAT diagnostic algorithms and treatment administration (including NECT), which often exceed the capacities of health centers and district hospitals in resource-constrained settings where HAT is endemic. Another impediment is the physical and logistical difficulties in reaching some of the affected populations.

A strong vertical component thus remains necessary for HAT surveillance and case management, particularly in areas where the disease is uncontrolled. Active case finding (including mass screening) for *T. b. gambiense* followed by treatment is a highly recommended control measure in such areas. Access to laboratory testing is necessary for screening and diagnosis, which involves resource-intensive procedures including lumbar punctures. Intervening in conflict zones to reach patients trapped by violence is a major challenge. Context-appropriate program approaches that take into account the complex epidemiology of HAT and the precarious situations in which it is found are still necessary. An example is described in Box 1.

**Box 1. NECT Implementation Experience–Example of MSF**

**DRC: HAT Treatment With NECT in a Conflict Zone**

Since its first HAT program in Uganda in 1986, MSF has treated close to 50,000 people for the disease over the last 25 years. As of the time of this writing, MSF currently runs HAT treatment programs in CAR, Chad, DRC, and Uganda. NECT has been implemented in these projects, with the first patient treated in Moissala, Chad in December 2009.

DRC has the highest endemicity for HAT [2], especially in certain hot spots around the country. From July 2007 to March 2009, MSF monitored and treated HAT in three districts (Doruma, Ango, and Bili) in the Haut-Uélé region of Orientale Province in northeastern DRC. In this program, 46,601 people were tested (18,559 passive screening and 28,042 active screening), of whom 1,570 were infected with *T. b. gambiense* (overall prevalence of 3.4%) and received treatment. Of those infected, 60% (947/1,570) were in the first stage of HAT [34]. Second-stage patients were treated with standard eflornithine monotherapy, which in this remote region required substantial logistical support and a significant effort to hire and train additional nursing staff.

In 2008, the rebel Lord's Resistance Army (LRA) scaled up their attacks against the local population; the level of violence was further escalated following a joint military operation launched against the LRA by the Congolese army and other armed groups. With insecurity high, in March 2009 following an attack on the MSF team, the program in Haut-Uélé was halted. Local health facilities did not have the logistical or human resources to maintain HAT diagnosis, treatment, or follow-up activities, leaving many patients without care. After the security situation improved, MSF returned to Doruma district in September 2009 to support the local hospital. HAT treatment activities resumed in January 2010, with the introduction of NECT for routine first-line treatment of second-stage *T. b. gambiense* HAT patients.

NECT's more practical and shorter treatment regimen was felt by MSF and its local partners as key for its feasibility of use, considering the paucity of nursing staff and the precarious security situation in Doruma. This also applies to other insecure areas where MSF currently conducts HAT control activities, such as in northern CAR [34].

## Limitations of NECT

NECT has a number of limitations as a treatment option for HAT. It is likely less effective against *T. b. rhodesiense* HAT, which badly needs different and better drugs for both stages of the disease. Administration of NECT is relatively complicated, including the requirement of two IV infusions per day for one week. Although this protocol is shorter and simpler than eflornithine monotherapy, and safer than melarsoprol, it is still resource- and training-intensive. Thus, a simpler regimen, preferably based on an oral drug formulation, is desirable. A treatment effective for both disease stages may eliminate the need for painful lumbar punctures and difficult examination of the cerebrospinal fluid, which are currently performed for HAT staging.

NECT's limitations highlight the ideal features desired in a new drug for HAT treatment [29]:

- Safe, low toxicity
- Oral, or noninjectable
- Effective in both HAT disease stages
- Effective against both types of trypanosomal parasites: *T. b. gambiense* and *rhodesiense*
- Short treatment duration
- Minimal training needs, readily implementable
- Affordable

## Discussion and Conclusions

The development, acceptance, and initial rollout of NECT have been exciting and emboldening advances for HAT treatment. Efficient NECT delivery must now be sustained to ensure this new therapeutic option reaches all patients in need.

Continuous wide-scale utilization of the toxic drug melarsoprol and of eflornithine in monotherapy, which may trigger parasitic resistance to this life-saving drug, highlight the urgency of replacing existing treatments with NECT for second-stage *T. b. gambiense* HAT.

Even if wide-scale NECT delivery is achieved, better drugs and diagnostics are still required to improve HAT control. R&D of new drugs is underway by a number of groups. In September 2009, DNDi entered a phase I clinical trial of a drug candidate given orally for HAT, fexinidazole [30]. Currently this is the only new drug candidate in clinical development for HAT. More drug compounds are needed in the R&D pipeline.

R&D for better diagnostic tools for HAT are also needed. The sensitivity of parasite detection tools in body fluids is currently limited [31], [32]. In addition, diagnosis of trypanosomal infection of the central nervous system requires a lumbar puncture, which is painful and difficult to perform, especially in resource-constrained settings. Field-adapted, rapid diagnostic tests for HAT diagnosis and staging must be developed if complete HAT control, including integration into primary health care centers, is to be feasible. The introduction of novel biomarkers, including recently identified markers for disease staging [33], and the development of field-adapted tests will require the mobilization of research laboratories with adequate funding.

Although there has been recent discourse that the elimination of HAT is feasible, this lofty goal is not likely to be possible in the near future given ongoing constraints, namely the difficulties of implementing complex diagnostic–treatment algorithms in resource-poor areas of high endemicity and persistent security threats. Even if perfect treatment and diagnostic tools were readily available for HAT, certain patient populations would still be difficult or impossible to reach.

HAT control in these hotspots should therefore be addressed through targeted programming and access, with robust surveillance and response. International donors and policymakers should be made aware that a “one size fits all” integrated approach may not be suitable for HAT in certain contexts and with the current tools. Dedicated funding for diagnosis and treatment and R&D, as well as allocated national program funding, must be put forth and sustained. The current paucity of international donors funding HAT control national programs is highly worrisome. Still and in the future, continued political pressure and will are needed for the prioritization of HAT patient care.
